# A mathematical look at empathy

**DOI:** 10.7554/eLife.47036

**Published:** 2019-04-29

**Authors:** Naoki Masuda, Francisco C Santos

**Affiliations:** 1 Department of Engineering MathematicsUniversity of BristolBristolUnited Kingdom; 2 Department of Computer Science and EngineeringInstituto Superior Técnico, Universidade de LisboaLisboaPortugal

**Keywords:** game theory, cooperation, social psychology, theory of mind, None

## Abstract

When an individual makes a judgement about the actions of another individual, taking the latter's viewpoint into consideration enhances cooperation in society at large.

**Related research article** Radzvilavicius AL, Stewart AJ, Plotkin JB. 2019. Evolution of empathetic moral evaluation. *eLife* **8**:e44269. doi: 10.7554/eLife.44269

The pros and cons of public and private transport are well known: public transport is more friendly to the environment and to society at large, but it can be inconvenient to the individual; cars and other forms of private transport, on the other hand, are convenient for individuals but are more harmful to the environment. The debate about public versus private transport is an example of a social dilemma that has fascinated psychologists, economists, mathematical biologists and many others for decades. In particular, how and why do humans (and other animals) cooperate and act in ways that put the interests of society at large ahead of their own interests and convenience?

Several mechanisms have been identified over the years to explain how cooperation is maintained when people are confronted with such social dilemmas (Sigmund, 2010). One explanation is that cooperation relies on a mechanism called 'indirect reciprocity' that is based on reputation: my decision to cooperate with you depends on your reputation. To illustrate this, consider the following example: Alice has to decide whether or not to help Bob. By helping Bob, Alice may improve her own reputation, and thus increase her chances of being helped by someone else in the future. Alternatively, if she decides not to help Bob, her reputation will be damaged, lowering her chances of being helped in the future.

Although the concept of reputation-based cooperation may sound intuitive, it is in fact more complex than it seems. First, we need to define what is meant by 'good' and 'bad'. For example, if Alice chooses to help Bob, but Bob is perceived to be a 'bad' person, should this result in a 'good' reputation? And if she decides not to help Bob (Figure 1), should this be seen as 'bad'? One can continue this line of thought and find the moral codes that allow cooperation to thrive, and show that few rules for assigning reputation are simple enough to appeal to intuition while also being able to promote cooperation (Ohtsuki and Iwasa, 2004; Ohtsuki and Iwasa, 2006; Santos et al., 2018).

**Figure 1.**
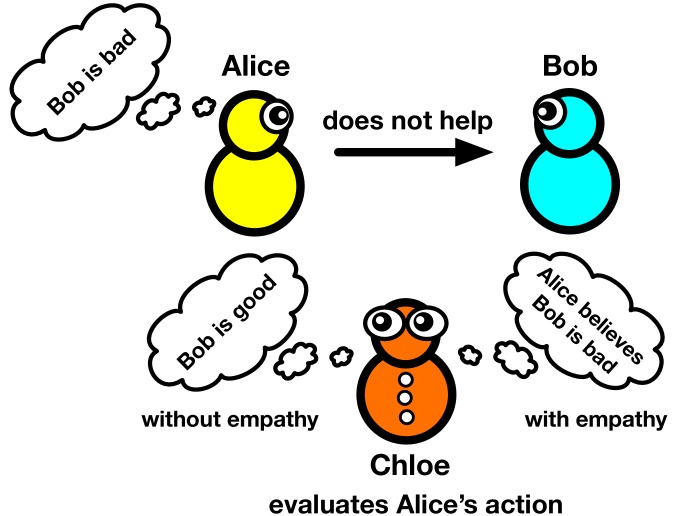
Reputation and empathy. Reputations have an important role in decisions to co-operate: for example, Alice will decide to cooperate with Bob if he has a good reputation, and decide to not cooperate if he has a bad reputation. Consider the case in which an observer (Chloe) witnesses Alice deciding not to help Bob because Alice believes that Bob is bad. In the absence of empathy (*E*=0; left), Chloe's opinion of Alice is based solely on the Chloe's existing opinion of Bob; that is, Chloe thinks Alice is bad because she thinks Bob is good. However, when Chloe has complete empathy for Alice (*E*=1; right), Chloe's opinion of Bob is based on Alice's opinion of Bob: that is, Chloe accepts Bob is bad because Alice thinks he is bad. Radzvilavicius et al. have explored the effect of empathy on co-operation when there is no consensus about reputations.

Second, the efficiency of these rules will depend on the information that is available to different people. Earlier mathematical models assumed that reputations are public, being instantly shared across society, but this is unlikely unless there is a central institution managing this information. It is more likely that different people will be able to have different opinions about reputations, making it more difficult to maintain cooperation (Uchida, 2010; Okada et al., 2017; Hilbe et al., 2018). Now, in eLife, Arunas Radzvilavicius and Joshua Plotkin of University of Pennsylvania, working with Alexander Stewart of University of Houston, report the results of mathematical modelling that offer new insights into the effect of empathy on cooperation when there is no consensus about reputations (Radzvilavicius et al., 2019).

In this context, empathy is the ability of someone to change their opinion of a person based on what other people think of that person (Radzvilavicius et al., 2019). Let us return to the example of Alice and Bob (Figure 1): Alice has chosen not to cooperate with Bob because she believes he is a 'bad' guy. A bystander, called Chloe, observes this action and, in the absence of empathy, she will assign Alice a bad reputation because, from her own perspective, she believes Bob to be good. Crucially, Radzvilavicius et al. included empathy – the possibility that Chloe may understand Alice’s point of view – in their model. The level of empathy E could range from zero (ie, Chloe has zero empathy with Alice) to one (ie, Chloe completely empathizes with Alice). Complete empathy would mean that Chloe thinks: "OK, although it is different from my opinion, Alice thinks Bob is a bad guy and I accept her view". In other words, Chloe has some 'theory of mind', understanding Alice’s intentions and perspective, even if they are different from her own. As a result, Chloe assigns Alice a good reputation because Alice has done the right thing according to Alice’s (not Chloe’s) point of view.

Radzvilavicius et al. conducted mathematical and numerical analysis to show that the empathy often enhances cooperation. Radzvilavicius et al. also showed that empathy itself is selected by evolution: if empathy is an individual property and is allowed to change over time through social learning (that is, through individuals mimicking other individuals who are successful), *E* often evolves towards larger values, leading to a more empathetic society.

Many questions, however, still remain. For instance, what are the mechanisms that enable an individual, such as Chloe, to know how a person’s reputation, such as Bob’s, is perceived by others? Secondly, if Chloe has more accurate information about Bob than Alice, how will this affect her empathy? Finally, does the structure of social networks (Newman, 2010) matter for how reputations spread in society? Empathy may also be seen as a form of tolerance and, in principle, be used to foster cooperation under the various and evolving moral codes that are typical of the world we live in. Overall, it may offer a new route towards a culture of tolerance, diversity and pro-sociality.
